# GEV*^Sod2^* Powder: A Modified Product Based on Biovesicles Functioned in Air Pollution PM2.5-Induced Cardiopulmonary Injury

**DOI:** 10.34133/research.0609

**Published:** 2025-02-13

**Authors:** Xiao Zhang, Xuan Ye, Yuling Xie, Zijiang Yang, Michail Spanos, Zilin Guo, YuXin Jin, Guoping Li, Zhiyong Lei, Raymond M. Schiffelers, Joost P. G. Sluijter, Hongyun Wang, Huihua Chen, Junjie Xiao

**Affiliations:** ^1^School of Traditional Chinese Medicine, Shanghai University of Traditional Chinese Medicine, Shanghai 201203, China.; ^2^ Joint International Research Laboratory of Biomaterials and Biotechnology in Organ Repair (Ministry of Education), School of Life Science, Shanghai University, Shanghai 200444, China.; ^3^ Institute of Cardiovascular Sciences, Shanghai Engineering Research Center of Organ Repair, School of Medicine, Shanghai University, Shanghai 200444, China.; ^4^ Department of Cardiovascular Surgery, Fujian Medical University Union Hospital, Fuzhou 350001, China.; ^5^ Fujian Provincial Center for Cardiovascular Medicine, Fuzhou 350001, China.; ^6^ Cardiovascular Division of the Massachusetts General Hospital and Harvard Medical School, Boston, MA, USA.; ^7^ QianWeiChang College, Shanghai University, Shanghai 200444, China.; ^8^ CDL Research, University Medical Center Utrecht, Utrecht, The Netherlands.; ^9^ Department of Cardiology, Laboratory of Experimental Cardiology, University Medical Center Utrecht, Utrecht, The Netherlands.; ^10^ UMC Utrecht Regenerative Medicine Center, Circulatory Health Research Center, University Medical Center, Utrecht University, Utrecht, The Netherlands.

## Abstract

The prevention of air pollution-related cardiopulmonary disorders has been largely overlooked despite its important burden. Extracellular vesicles (EVs) have shown great potential as carriers for drug delivery. However, the efficiency and effect of EVs derived from different sources on ambient fine particulate matter (PM2.5)-induced cardiopulmonary injury remain unknown. Using PM2.5-exposed cellular and mouse models, we investigated the prevention of air pollution-related cardiopulmonary injury via an innovative strategy based on EV delivery. By using a “2-step” method that combines bibliometric and bioinformatic analysis, we identified superoxide dismutase 2 (*Sod2*) as a potential target for PM2.5-induced injury. *Sod2*-overexpressing plasmid was constructed and loaded into human plasma-, bovine milk-, and fresh grape-derived EVs, ultimately obtaining modified nanoparticles including PEV*^Sod2^*, MEV*^Sod2^*, and GEV*^Sod2^*, respectively. GEV*^Sod2^*, especially its lyophilized GEV*^Sod2^* powder, exhibited superior protection against PM2.5-induced cardiopulmonary injury as compared to PEV*^Sod2^* and MEV*^Sod2^*. High-sensitivity structured illumination microscopy imaging and immunoblotting showed that GEV*^Sod2^* powder treatment altered lysosome positioning by reducing Rab-7 expression. Our findings support the use of fruit-derived EVs as a preferred candidate for nucleic acid delivery and disease treatment, which may facilitate the translation of treatments for cardiopulmonary injuries.

## Introduction

Cardiovascular disease (CVD) represents a significant threat to human lifespan and is closely associated with all-cause mortality in patients with chronic respiratory disease (CRD). Notably, exposure to ambient air pollutants has been identified as a major factor contributing to the increased mortality rates associated with both CVD and respiratory disorders (RPD), such as ischemic heart disease, chronic obstructive pulmonary disease, and even lung cancer [1]. According to the World Health Organization, air pollution is responsible for 13 deaths per minute, primarily due to heart diseases, strokes, and lung cancer. Among air pollutants, fine particulate matter (PM2.5) significantly exacerbates the burden of cardiopulmonary diseases. Evidence increasingly suggests that both low and long/short-term exposures to PM2.5, a primary contributor to atmospheric haze, correlate with increased mortality and cardiopulmonary risk [2–4] as well as systemic adverse alterations, including inflammation, oxidative stress, and pathological disorders [5,6]. Long-term exposure to PM2.5, following a 3.7 μg/m^3^ increment in PM2.5 levels, has been linked to over a 10% increase in nonrespiratory infection-related hospital admissions [7]. Several strategies have been developed for treating different cardiovascular or vascular dysfunction [8–10]; however, interventions for air pollution-related cardiopulmonary injuries have been largely neglected in clinical settings due to their nearly invisible and nondetectable characteristics. Thus, finding innovative effective interventions for CVD and RPD, based on ambient PM2.5-induced cardiopulmonary injury, is crucial.

Extracellular vesicles (EVs), nanoparticles secreted by nearly all cell types and present in biofluids, act as biological couriers, encapsulating various biomolecules (DNA, RNA, proteins, and metabolites) and mediating inter-organ communication, thereby participating in numerous physiological and pathological processes [11,12]. EVs offer unique advantages in drug delivery as natural molecular cargos, with their utility in disease treatment as demonstrated by their promotion of repair and improvement of organ functions [13]. Recently, plant-derived EV-like nanoparticles, particularly those from edible plants like grapes, have gained attention for their therapeutic potential [14], and serving as potential treatments for vascular calcification [15]. Although a multitude of EV sources have been suggested to mediate functional beneficial effects, a direct comparison among them for different functional efficiencies still requires further clarification. Here, we aimed to identify which source-derived EVs are best suited for delivering biologically active molecules to mitigate PM2.5-induced cardiopulmonary stress. In addition, we defined conditions and methods for storing EVs to maintain their biological efficacy.

In this study, we investigated 3 potential biological fluids that are relatively easily obtained and have previously been shown to be effective (human plasma, bovine milk, and fresh grape juice), and we explored which source is most effective for delivering an antioxidant *Sod2*-overexpressing plasmid. We compared the biological characteristics, therapeutic effects, and immunogenicity of these EVs in a PM2.5-exposed model, including acute in vivo exposure (intratracheal instillation) and in vitro human bronchial epithelial cell and cardiomyocyte models. Upon defining a bacterial- and additive-free EV powder, we evaluated the impact on acute PM2.5-induced cardiopulmonary injury based on comparisons in Beas-2B cells and primary neonatal rat cardiomyocytes (NRCMs), which included oxidative stress, inflammation, lysosomal positioning, and cell apoptosis.

## Results

### Sod2 is decreased in acute PM2.5 exposure-induced cardiopulmonary injury

**Fig. 1.**
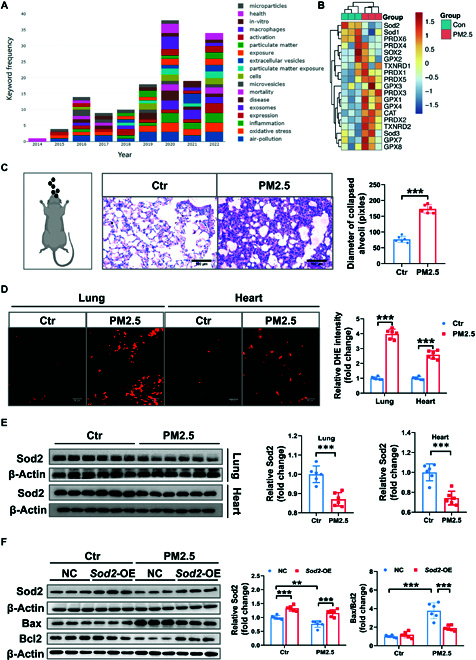
Sod2 is decreased in PM2.5-induced acute cardiopulmonary injury*.* (A) Keyword frequency analysis of published literatures in PM2.5-related pulmonary and cardiovascular disorders by bibliometrics. (B) Heat map of oxidative stress genes in filtered air and PM2.5-exposed mice by bioinformatics. (C) Schematic diagram of PM2.5 intratracheal instillation (left panel) and the representative images of H&E staining of lung tissue sections (middle panel) and quantification of collapsed alveolar diameter (right panel). (D) DHE staining of lung and heart tissues in PM2.5- or PBS-exposed mice (Ctr, *n* = 6 versus PM2.5, *n* = 6). (E) The protein content of Sod2 was tested in the mouse lungs and hearts (control, *n* = 6 mice versus PM2.5, *n* = 6). (F) The protein expression level of Sod2, Bax, and Bcl2 was tested in Beas-2B cells with FUGW-*Sod2*-overexpressing (OE) or control plasmid (NC) transfection under PBS or PM2.5 exposure. β-Actin served as control (*n* = 6). Data were shown as means, and error bars indicate the SD (means ± SD). *P* values were calculated with the unpaired, 2-tailed Student’s *t* test (C to E) after passing normality test between the Ctr and PM2.5 group. To compare multiple groups, 2-way ANOVA test with Tukey’s post hoc test was used (F).

Oxidative stress is extensively studied in numerous diseases and dysfunctions. To elucidate the whole role of oxidative stress in PM2.5 toxicity, we conducted a bibliometric analysis of published literature using keywords such as “particulate matter”, “cardiovascular”, “heart”, “exosomes”, “extracellular vesicles”, and “lung”. Analysis through online bibliometries platform showed that “inflammation” and “oxidative stress” were among the high-frequency keywords (Fig. 1A), indicating these processes as likely underlying central mechanisms of PM2.5-induced injury. Through subsequent bioinformatic analysis of RNA-sequencing data from mice exposed to filtered air versus PM2.5 [National Center for Biotechnology Information (NCBI) Sequence Read Archive database, PRJNA540011], we observed a reduction in superoxide dismutase 2 (Sod2) expression following PM2.5 exposure (Fig. 1B), suggesting a potential regulatory role of oxidative stress involved *Sod2* in air pollution-related lung injury. Subsequently, we performed experimental verification in vivo and in vitro. Given the ongoing improvements in air quality but sometimes with sudden moderate to severe pollution, we utilized a model of short-term rather than long-term acute exposure via intratracheal instillation to assess the oxidative stress and apoptotic changes in lung and heart tissues of mice. Hematoxylin and eosin (H&E) staining showed that PM2.5 exposure led to thickening of the airway epithelium, enlargement of alveolar spaces, and collapse of alveoli, with a notable increase in alveolar cavity size (Fig. 1C). Dihydroethidium (DHE) staining indicated a significant increase in reactive oxygen species (ROS) production in the lungs and heart tissues (Fig. 1D) in the PM2.5-exposed group, confirming that PM2.5 exposure markedly induces oxidative stress, consistent with previous findings [6]. Western blot analysis showed more than a 2-fold increase in Bax/Bcl2 and cleaved caspase-3/caspase-3 in the lungs (Fig. S1A) and a near doubling in the heart tissues compared to control groups (Fig. S1B). These results demonstrate that acute exposure to PM2.5 can induce cardiopulmonary injury, characterized by oxidative stress and cell apoptosis, ultimately leading to pathological structural tissue changes.

We assessed the mRNA and protein levels of Sod2 in lung and heart tissues and could confirm that PM2.5 exposure resulted in a significant decrease in *Sod2* mRNA level (Fig. S2A and B) and protein expression (Fig. 1E) in both organs. To investigate the direct effect of Sod2 in PM2.5-induced cell apoptosis further, we transfected Beas-2B cells with a *Sod2*-expressing plasmid prior to PM2.5 treatment. Upon transfection, *Sod2*-expressing plasmid transfection resulted in an increase in the protein expression as compared to the control group under basal and PM2.5 conditions (Fig. 1F). To assess the effect of Sod2 in PM2.5-induced cell apoptosis, we tested the protein expression of apoptotic-related marker. Our data showed that overexpression of Sod2 effectively down-regulates Bax and up-regulates Bcl2 expression (Fig. 1F), indicating that Sod2 serves as a protective anti-oxidative enzyme against PM2.5-induced cellular toxicity.

### Grape-derived EVs preferably load *Sod2* to combat PM2.5-induced cell oxidative stress, inflammation, and apoptosis

**Fig. 2.**
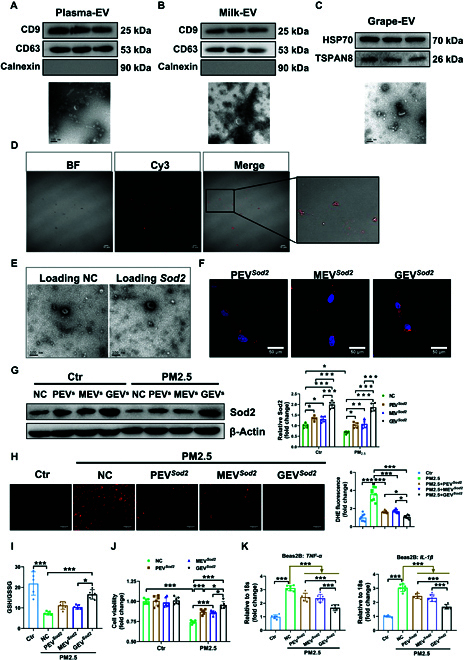
Grape-derived EVs preferably load *Sod2* to combat PM2.5-induced cell oxidative stress, inflammation, and apoptosis. EVs derived from (A) human plasma (*n* = 3), (B) milk (*n* = 3), and (C) fresh grape juice (*n* = 3) were characterized by protein marker expression (top panel). Morphology identification of EVs was examined by TEM (bottom panel). (D and E) Cargo loading and morphology observation upon fresh grape juice-derived EVs. EVs from human plasma, milk, and fresh grape juice were loaded with *Sod2*-expressing plasmid, which were labeled as PEV*^Sod2^*, MEV*^Sod2^*, and GEV*^Sod2^* respectively. (D) Fluorescence images of Cy3-siRNA (small interfering RNA) NC after loading into EVs. (E) Morphology of NC- and *Sod2*-expressing plasmid-loading EVs by TEM. (F) Cellular uptake of PEV*^Sod2^*, MEV*^Sod2^*, and GEV*^Sod2^* was tested by immunofluorescence imaging. (G) Sod2 was examined by Western blotting. β-Actin was used as the control (*n* = 5). The cell viability and inflammatory factor content of Beas-2B cells pretreated with PEV*^Sod2^*, MEV*^Sod2^*, and GEV*^Sod2^* in PM2.5 mode were evaluated by (H) DHE staining (*n* = 8), (I) cellular GSH/GSSG level (*n* = 5), (J) cell counting kit 8 (CCK8) test, and (K) real-time PCR test of inflammatory factors (*n* = 6).

Given that increasing the expression of Sod2 can confer resistance to PM2.5-induced toxicity, identifying effective methods to promote the expression of Sod2 is critical. Currently, various methods for gene expression regulation are available, including gene editing, adeno-associated virus vector (AAV)-based gene therapy, and lipid nanoparticle-based delivery. However, these methods have been shown to have varying degrees of side effects [16–18]. In recent years, EVs have emerged as a promising tool for drug delivery due to their unique properties. Notably, an increasing number of studies have highlighted the relationship between exosomes and inflammation, particularly regarding their encapsulated contents [19]. Here, we aimed to identify a safe source of EV cargo for drug delivery purposes. Considering factors such as cost, easy availability, and potential sensitization, we selected mammalian milk and edible plant juice (grape) as initial materials for EVs isolation. We also included human plasma-derived EVs for comparison in the PM2.5 model, building further upon our previous study [20]. As shown in Fig. 2, EVs derived from human plasma, milk, and fresh grape juice were characterized by protein marker expression, nanoparticle tracking analysis, and morphological identification. Plasma- and milk-derived EVs exhibited typical characteristics, expressing protein markers CD9 molecule (CD9) and CD63 molecule (CD63) while lacking negative marker calnexin (Fig. 2A and B). Significant expression of heat shock protein 70 (HSP70) and tetraspanin 8 (TSPAN8) was observed in grape-derived EVs (Fig. 2C). Moreover, transmission electron microscopy (TEM) images confirmed that EVs from plasma, milk, and grape all displayed the classic spherical morphology and containing membrane-enclosed structures (Fig. 2A to C). Interestingly, the average size of these 3 types of EVs ranged between 100 and 150 nm, with no significant difference between plasma- and milk-derived EVs (Fig. S3A and B). Notably, grape-derived EVs were slightly larger than those derived from mammalian sources, exhibiting slight increases in size compared to plasma- and milk-derived EVs, respectively.

Prior to preparing *Sod2* loading EVs, we assessed the loading efficiency using electrorotation with Silencer Cy3-labeled Negative Control siRNA (Fig. 2D) and examined the morphology of freshly isolated EV loading negative control (NC) or *Sod2*-expressing plasmid via TEM (Fig. 2E), noting no significant morphological differences between NC and *Sod2* loading operation.

To assess the efficacy of *Sod2*-loaded plasma-derived (PEV*^Sod2^*), milk-derived (MEV*^Sod2^*), and grape-derived (GEV*^Sod2^*) EVs against PM2.5-induced cell toxicity, Beas-2B cells were cultured to examine changes in cell viability, oxidative stress, apoptotic flux, and inflammatory factor release in vitro. 1,1′-Dioctadecyl-3,3,3′,3′-tetramethylindodicarbocyanine, 4-chlorobenzenesulfonate salt (DiD) labeling showed that PEV*^Sod2^*, MEV*^Sod2^*, and GEV*^Sod2^* were all successfully taken up by the cells (Fig. 2F) and GEV*^Sod2^* displayed a higher Sod2 expression than did PEV*^Sod2^* and MEV*^Sod2^* in PM2.5-exposed Beas-2B cells (Fig. 2G). Specifically, GEV*^Sod2^* significantly enhanced the anti-oxidative capacity of cells, reducing the ROS levels (Fig. 2H) and increasing the GSH [glutathione (reduced form)]/GSSG (oxidized glutathione) ratio from 7.46 to 16.59 (Fig. 2I). In addition, GEV*^Sod2^* exhibited a stronger capacity to prevent the reduction of cell viability after PM2.5 exposure, from 74% (control group) to 95% (Fig. 2J).

In recent years, EVs’role in inflammation has attracted increasing attention. Thus, the impact of these modified EVs on the secretion of inflammatory factors was assessed using Beas-2B and human cardiomyocyte AC16 cells to establish a PM2.5 in vitro model. Exposure to PM2.5 significantly reduced *IL-10* levels while increasing *TNF-α*, *IL-1β*, and *IL-6* levels in Beas-2B cells (Fig. 2K and Fig. S4A and B). Interestingly, PEV*^Sod2^*, MEV*^Sod2^*, and GEV*^Sod2^* could increase *IL-10* expression by 69.7%, 79.1%, and 88.4%, respectively (Fig. S4A), and simultaneously suppress *TNF-α* and *IL-1β* expression after PM2.5 exposure in Beas-2B cells. Hereby, GEV*^Sod2^* exhibited the strongest effect in reducing *TNF-α* and *IL-1β* levels by 46.47% and 44.4%, respectively (Fig. 2K). However, *IL-6* levels were not altered in the PEV*^Sod2^* and MEV*^Sod2^* groups, while these were significantly reduced by 35.2% in the GEV*^Sod2^*-treated group as compared to the control group (Fig. S4B). Similar trends were observed in human AC16 cells, with GEV*^Sod2^* showing an increase in *IL-10* expression and a decrease in *TNF-α*, *IL-1β*, and *IL-6* levels (Fig. S4C to F). Taken together, these data indicate that GEV*^Sod2^* offers superior protection against cell death, oxidative stress, and the release of inflammatory factors upon PM2.5 exposure.

### GEV*^Sod2^* powder (DP-GEV*^Sod2^*) treatment significantly reduced PM2.5 toxicity

**Fig. 3.**
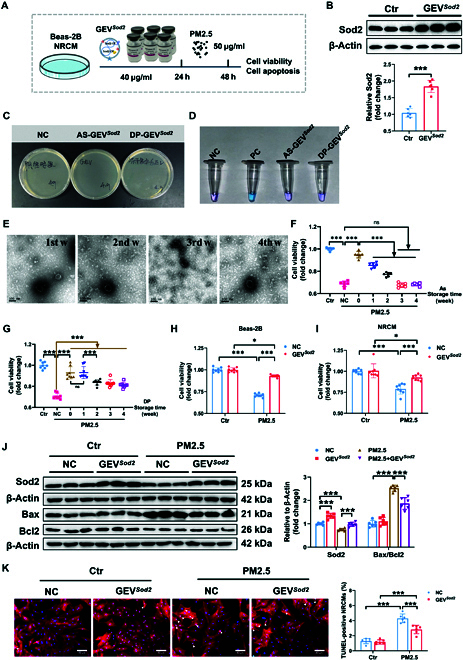
GEV*^Sod2^* powder (DP-GEV*^Sod2^*) treatment significantly reduced PM2.5 toxicity. (A) Flowchart of the therapeutical experiment in vitro. (B) Representative Western blots for the Sod2 protein level in the control or GEV*^Sod2^*-treated Beas-2B cells (Ctr, *n* = 6 versus GEV*^Sod2^*, *n* = 6). (C) Bacterial and (D) mycoplasma detection of AS-GEV*^Sod2^* and DP-GEV*^Sod2^*. (E) The morphology of DP-GEV*^Sod2^* stored at −80 °C for 1, 2, 3, or 4 weeks was observed by TEM. (F) The effect of cryopreserved aqueous solution GEV*^Sod2^* (AS-GEV*^Sod2^*) on PM2.5-exposed cell viability at 1, 2, 3, and 4 weeks was tested by CCK8 (*n* = 6). (G) Effect of cryopreserved GEV*^Sod2^* powder (DP-GEV*^Sod2^*) on PM2.5-exposed cell viability at 1, 2, 3, and 4 weeks (*n* = 8). The efficiency of DP-GEV*^Sod2^* was assessed in pre-PM2.5-exposed (H) Beas-2B and (I) NRCM cells by testing cell viability test. (J) The expression of Sod2, Bax, and Bcl2 protein in the NC, GEV*^Sod2^*, PM2.5, and GEV*^Sod2^* + PM2.5-treated Beas-2B cells was tested by Western blots (*n* = 6). (K) TUNEL/α-actinin fluorescent staining in NRCM with GEV*^Sod2^* or NC treatment after PBS or PM2.5 exposure (*n* = 6). Data are shown as means ± SD. *P* values were calculated with the unpaired, 2-tailed Student’s *t* test (B) after passing normality test. To compare multiple groups, 1-way ANOVA (F and G) and 2-way ANOVA with Tukey’s post hoc test was used (H to K).

To further confirm the therapeutical effect of GEV*^Sod2^* after PM2.5 exposure, we performed a treated experiment as depicted in Fig. 3A. Previous research has demonstrated that storing EVs in phosphate-buffered saline (PBS) results in a significant reduction of recovery even within days [21], emphasizing the importance of optimal storage conditions to preserve EV biological activity. Motivated by conventional reagent storage techniques, we aimed to stabilize the activity of aqueous solution (AS-GEV*^Sod2^*) by converting it into a powder form (DP-GEV*^Sod2^*) using vacuum freeze-drying. First, we employed Western blot analysis to evaluate the efficacy of DP-GEV*^Sod2^* in delivering *Sod2* to cells and observed successful enhancement of Sod2 protein expression in Beas-2B cells by GEV*^Sod2^* treatment (Fig. 3B). Before using this product, we examined the safety of DP-GEV*^Sod2^* by performing bacterial and mycoplasma tests, finally confirming that DP-GEV*^Sod2^* was sterile and free from mycoplasma contamination (Fig. 3C and D).

To compare the longevity of AS-GEV*^Sod2^*’ and DP-GEV*^Sod2^*’s activity when stored at −80 °C, we conducted a time-series study including evaluating functionality. Time-course studies showed that DP-GEV*^Sod2^* maintained a relatively stable morphology after 4 weeks of storage at −80 °C (Fig. 3E). The protective role of AS-GEV*^Sod2^* at −80 °C diminished significantly with 10% within 1 week and 18.7% within 2 weeks. Accordingly, AS-GEV*^Sod2^* lost its protective capacity entirely when stored over 2 weeks in the PM2.5-exposed cell model (Fig. 3F). DP-GEV*^Sod2^* demonstrated superior efficacy in enhancing cell viability after PM2.5 exposure for up to 4 weeks. Specifically, DP-GEV*^Sod2^* maintained cell viability from 70.7% to 93% within the first week (Fig. 3G) and was still able to protect Beas-2B cells from PM2.5-induced toxicity after 4 weeks, although with slight functional reduction.

To further confirm the therapeutical effect of GEV product, Beas-2B cells and NRCM were cultured and exposed to PM2.5 prior to GEV*^Sod2^* treatment for 24 h. Beas-2B and NRCM cells receiving GEV*^Sod2^* treatment after PM2.5 exposure showed a higher cell viability than did the GEV*^Sod2^*-free group (Fig. 3H and I). Consistent with cell viability analysis, Beas-2B cells receiving GEV*^Sod2^* treatment after PM2.5 exposure kept a solid protein expression of Sod2, contributing to a reduced Bax/Bcl2 ratio compared to the GEV*^Sod2^*-free group (Fig. 3J). We treated NRCM with DP-GEV*^Sod2^* under PM2.5 exposure, resulting in a reduced number of TUNEL (terminal deoxynucleotidyl transferase–mediated deoxyuridine triphosphate nick end labeling)-positive NRCM cells following PM2.5 exposure as compared to the control group (Fig. 3K).

These findings demonstrate that DP-GEV*^Sod2^* is more suitable than AS-GEV*^Sod2^* for low-temperature storage and can effectively protect pulmonary cells and cardiomyocytes against PM2.5-induced cell toxicity.

### GEV*^Sod2^* product (DP-GEV*^Sod2^*) significantly alleviates acute PM2.5 exposure-induced lung injury

**Fig. 4.**
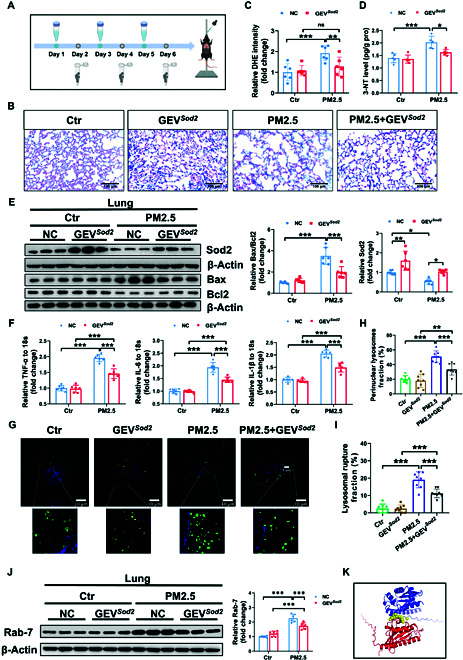
DP-GEV*^Sod2^* significantly alleviates acutes PM2.5-induced pulmonary oxidative stress, inflammation, lysosome positioning, and cell apoptosis. (A) Experimental diagram of PM2.5 exposure and DP-GEV*^Sod2^* treatment. DP-GEV*^Sod2^* was dissolved with PBS at a dose of 40 μg/ml prior to usage. (B) H&E staining of mouse lungs after PM2.5 exposure and DP-GEV*^Sod2^* treatment. Evaluating the oxidative stress content by (C) DHE staining (*n* = 6 lungs) and (D) 3-NT ELISA assay (*n* = 5 lungs). (E) The protein expression of Sod2, Bax, and Bcl2 was assessed after DP-GEV*^Sod2^* treatment in PM2.5 model. β-Actin was used as the control (*n* = 6). (F) Expression of *TNF-α*, *IL-6*, and *IL-1β* mRNA in PBS- and PM2.5-exposed mice lungs (NC, *n* = 6 versus DP-GEV*^Sod2^*, *n* = 6). 18*S* was used as an internal control. (G) Representative images of HIS-SIM staining after DP-GEV*^Sod2^* treatment in PM2.5-exposed model, and (H) quantification of perinuclear lysosome ratio (*n* = 8) and (I) lysosomal rupture fraction ratio (*n* = 8). (J) Expression of Rab-7 protein in the NC, GEV*^Sod2^*, PM2.5, and GEV*^Sod2^* + PM2.5 mouse lungs. β-Actin was used as the control (*n* = 6). (K) Predicted interaction map between human SOD2 (blue) and RAB7 (red). The predicted interactive amino acids were shown in yellow. Data were shown as means ± SD. To compare multiple groups, 2-way ANOVA test with Tukey’s post hoc test was used (C to F and H to J).

Observing the protective role of DP-GEV*^Sod2^* in vitro, we subsequently evaluated its efficacy in vivo using a PM2.5-induced acute lung injury model. The intervention flowchart for DP-GEV*^Sod2^* in the PM2.5 exposure model is shown in Fig. 4A. Mice received intratracheal instillation of DP-GEV*^Sod2^* thrice weekly, followed by PM2.5 exposure. Subsequently, pulmonary structure, oxidative stress, apoptotic, and inflammatory levels were assessed using H&E staining, enzyme-linked immunosorbent assay (ELISA), Western blotting, and real-time quantitative polymerase chain reaction (RT-qPCR), respectively. Our results showed that PM2.5 exposure led to alveolar collapse and dilatation, whereas DP-GEV*^Sod2^* treatment protected against these pathological alterations (Fig. 4B), indicating significant improvement in PM2.5-induced pulmonary pathology. Moreover, DP-GEV*^Sod2^* effectively reduced the stimulated DHE and 3-nitrotyrosine (3-NT) levels (Fig. 4C and D). Beyond its protective role in resisting oxidative stress, DP-GEV*^Sod2^* also demonstrated anti-apoptotic effects, enhancing the expression of Sod2 and Bcl2, and reducing the protein expression of Bax in lung tissues (Fig. 4E). PM2.5 significantly increased the levels of *TNF-α*, *IL-6*, and *IL-1β*, while DP-GEV*^Sod2^* treatment reduced their expression (Fig. 4F).

Previous research has demonstrated that lysosomal disorder is involved in PM2.5-induced ferroptosis and oxidative regulation [22], and the reduction of Sod2 contributes to lysosomal membrane permeabilization [23]. To investigate whether DP-GEV*^Sod2^* affected lysosomal homeostasis, we examined lysosome positioning and membrane integrity using high-sensitivity structured illumination microscopy (HIS-SIM) imaging. PM2.5 exposure resulted in perinuclear lysosome clustering and membrane destruction in Beas-2B cells, while DP-GEV*^Sod2^* treatment significantly shifted lysosome positioning from the perinuclear region to the peripheral space and alleviated lysosomal membrane damage (Fig. 4G to I). Consistent with previous findings, lysosome positioning can be influenced by Rab-7/RILP [24], and exposure to polystyrene nanoplastics can elevate cellular Rab-7 levels [25]. We evaluated the protein expression of Rab-7 in PM2.5-exposed Beas-2B cells and mouse lungs. Our data revealed that the Rab-7 protein was increased after PM2.5 exposure but significantly reduced following DP-GEV*^Sod2^* treatment (Fig. 4J). To investigate the possible mechanism of Sod2 regulating Rab7, molecular docking and potential interacting sites were predicted. As shown in Fig. 4K, the visualized map of human SOD2 (blue) and RAB7 (red) was produced by PyMOL Molecular Graphics System (version 3.0, Schrödinger LLC) based on the predicting data of AlphaFold Server [26]. The predicted interactive amino acids were shown in yellow, including Ser^27^, Asp^30^, Leu^31^, Pro^32^, Tyr^33^, Asp^34^, Tyr^35^, and Pro^107^.

In conclusion, DP-GEV*^Sod2^* can ameliorate PM2.5-induced pulmonary injury by improving alveolar structure, mitigating oxidative stress, reducing inflammatory factor expression, modifying lysosome positioning, and preserving lysosomal membrane integrity, thereby reducing cell apoptosis and tissue disorganization.

### GEV*^Sod2^* product improves acute PM2.5 exposure-induced cardiac oxidative stress, apoptosis, and inflammation

**Fig. 5.**
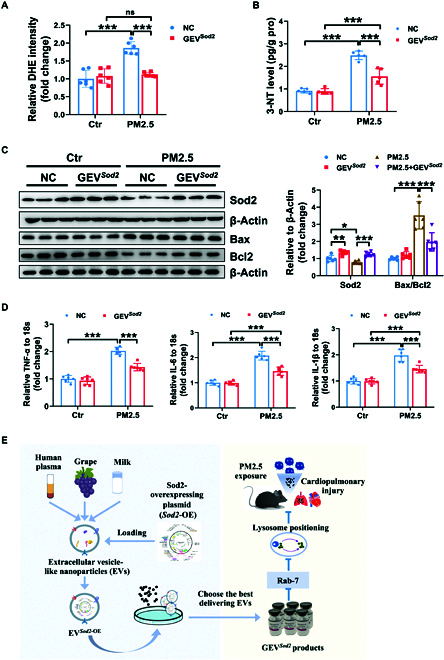
DP-GEV*^Sod2^* improves acute PM2.5-induced cardiac dysfunction. (A) Fluorescence intensity of DHE staining in PM2.5-exposed heart tissues, with NC or DP-GEV*^Sod2^* treatment (*n* = 6). (B) 3-NT level in the NC or DP-GEV*^Sod2^*-treated hearts, with PBS or PM2.5 challenge (*n* = 5). (C) The protein expression of Sod2, Bax, and Bcl2 was assessed after DP-GEV*^Sod2^* treatment in PM2.5 model. β-Actin was used as a control (*n* = 6). (D) Expression of *TNF-α*, *IL-6*, and *IL-1β* mRNA in PBS- and PM2.5-exposed mice hearts (NC, *n* = 6 versus DP-GEV*^Sod2^*, *n* = 6). 18*S* was used as an internal control. (E) Graphical abstract of this study. Data are shown as means ± SD. To compare multiple groups, 2-way ANOVA test with Tukey’s post hoc test was used (A to D).

Acute exposure to PM2.5 can lead to cardiac oxidative stress and inflammation, which precede functional and homeostatic imbalances. To assess the effect of DP-GEV*^Sod2^* on the heart, we monitored the flux of ROS and 3-NT, levels of apoptosis, and the content of inflammatory factors. Our data showed that DP-GEV*^Sod2^* significantly reduced the fluorescence intensity of DHE and the level of cardiac 3-NT under PM2.5 exposure conditions (Fig. 5A and B). Protein expression levels of Sod2 and Bcl2 were significantly elevated after DP-GEV*^Sod2^* treatment, compared to the control group in heart tissue lysates (Fig. 5C). Inflammatory factor levels, such as *TNF-α*, *IL-6*, and *IL-1β*, were also significantly reduced in the DP-GEV*^Sod2^* treatment group compared to controls after PM2.5 exposure (Fig. 5D).

In summary, DP-GEV*^Sod2^* is protective by reducing oxidative stress, cellular apoptosis, and inflammation induced by acute PM2.5 exposure in the lungs and hearts. The graphical abstract of this study is shown in Fig. 5E.

## Discussion

In the current work, we developed a “2-step” analysis method to identify the key gene *Sod2*, involved in PM2.5-induced cardiopulmonary injury, constructed an overexpressing plasmid, and delivered it to mice using naturally derived EVs. By comparing the anti-inflammatory, anti-oxidative, and anti-apoptotic capacities in PM2.5-exposed models, we determined that grape-derived EVs, GEV*^Sod2^*, were the most effective carriers, significantly reducing PM2.5-induced cardiopulmonary injury. Remarkably, GEV*^Sod2^* treatment substantially improved perinuclear lysosome clustering following PM2.5 exposure. Our findings suggest that plant-derived EVs are effective delivery carriers due to their protective capacities against oxidative stress and inflammation, thereby reinforcing the development of natural food-based therapeutic strategies.

EVs are natural biological messengers and have been thoroughly studied and utilized for delivering molecules and medicines. Despite studies on various biological fluids, including blood-, cell supernatant-, milk-, and plant-derived EVs, it remains unclear which source is most optimally effective for therapeutic molecule loading. In this study, we extracted EVs from human plasma, cow milk, and grapes, assessing their efficacy in delivering *Sod2*-overexpressing plasmid against PM2.5-induced stress, inflammation, and apoptosis. We found that GEV*^Sod2^* had superior effects in mitigating oxidative stress, inflammation, and cell toxicity. Overall, we recommend using natural safety fruits such as grapes as a source of EV delivering tools.

In the past few years, several studies showed that vegetables and fruit-derived exosome-like nanoparticles (ELNs) could reduce inflammation by transferring substances such as lipids, proteins, and miRNAs [14]. Especially, these 2 kinds of ELNs showed great performance in their antioxidative and antivirus activity [14]. However, there is no systematic comparison between vegetable- and fruit-derived ELNs. We cannot ignore some superiorities of vegetable-derived ELNs over that of grape; however, the main reasons that we chose grape as a resource of ELNs extraction include the following: First, fruit such as grape has peels and the removal of the peel may largely reduce the potential damage of microorganism. In addition, the grape has preferred antioxidative activity because of their enriched anthocyanin and glutathione.

Also, in this study, we addressed the challenge of EV storage, which is one of the identified limitations of their clinical use [27]. While trehalose has been suggested as an alternative for EV preservation [28], its potential to increase hypervirulence cannot be overlooked [29], and developing a method to store EVs without additives would be safer. For this reason, we used a straightforward procedure to create EV powder, enhancing EV stability during explored strategies to cryopreservation.

The interdependence of heart and lung diseases suggests that interventions for respiratory system exacerbations might also prevent adverse cardiovascular outcomes [30]. Previous studies indicate that cardiac damage can be secondary to lung injury, a consequence of organ crosstalk [31]. A prior study emphasized that chronic obstructive pulmonary disease increases cardiovascular event risk in a time-dependent manner [32], underscoring the potential of preventing cardiovascular dysfunction by mitigating lung damage. Endothelial cells may play important roles in the cross-talk between lungs and heart [33]. Consequently, we explored strategies to alleviate cardiopulmonary dysfunction, focusing on shared or interactive mechanisms. Here, we utilized an anti-oxidative enzyme delivered via intratracheal installation of EVs, significantly reducing apoptosis, oxidative stress, and inflammation in both heart and lung tissues. Notably, alternative methods such as nebulization inhalation have been investigated to administer drugs to mice, which may be preferred for aerosol administration [34].

Oxidative stress, implicated in numerous diseases, remains a critical subject in air pollution-associated cardiopulmonary injury research [35,36]. Supplementing antioxidants or increasing the expression of antioxidative enzymes can reduce oxidative stress, thereby protecting cells from oxidative damage. Our previous research showed that antioxidants like N-acetylcysteine (NAC) could significantly counteract PM2.5-induced damage in the lungs and heart [37]. Here, we have established a link between PM2.5-induced oxidative stress, cell death, and reduced Sod2 expression, marking *Sod2* as a potential therapeutic target for PM2.5-induced cardiopulmonary injury. Therefore, we conducted a series of experiments to identify a suitable natural carrier for delivering *Sod2*-overexpressing recombinant vector. Our findings reveal that GEV*^Sod2^* offers superior protection against PM2.5-induced cell injury, potentially due to a modest enhancement of Sod2 expression. Certainly, mRNA drugs or vaccines attracted a lot of attention as their superiorities in saving time and expense, being easy to design and produce. mRNA-based therapy showed promising effects such as rare disease [38], which is also within our scope in the future.

Here, we confirmed the protective impact of plant-derived nanoparticles, underscoring their viability as natural drug delivery systems due to their safety. The simplicity of production, low cytotoxicity, and excellent biocompatibility make plant-derived nanoparticles increasingly appealing. Notably, food-derived EVs have shown potential in treating diseases like tumors and vascular calcification [15,39]. Research from over a decade ago confirmed that fruit ELNs could shield mice from colitis [40]. Yet, the specific components of grape-derived particles contributing to their therapeutic potential remain unidentified. Given the known antioxidative properties of grape seeds and skins, we isolated EVs from grapes following the removal of these components, hypothesizing that bioactive substances within the juice, such as tartaric acid, l-malic acid, and microelements [41], might contribute to GEV^Sod2^’s efficacy against PM2.5.

Lysosome positioning is closely linked to cellular activity and energy metabolism and plays a vital role in regulating cellular nutrient conditions. Lysosome function is crucial in several pathologies such as breast cancer, immunity, neurological diseases, and cardiac fibrosis [42]. While a large number of studies have investigated lysosome positioning in energy deprivation and pathological conditions, few have focused on its association with PM2.5-induced respiratory dysfunction. Utilizing high-resolution SIM, we observed that PM2.5 exposure leads to notable perinuclear lysosome clustering and lysosomal membrane destruction. Conversely, GEV*^Sod2^* treatment facilitated the peripheral distribution and reduced the destruction of lysosomes. 

As an important regulator of lysosomal positioning and biological processes such as pro-apoptosis, the mechanism of Rab7 in the endocytic process has been extensively studied and overviewed. PARK8 (leucine-rich repeat kinase 2) was identified to localize to the lysosomal membranes and interact with Rab7, negatively regulating the perinuclear localization of lysosome [43]. In addition, other genes including ARL8B, KIF2A, and protrudin were identified to promote lysosome positioning from perinuclear to peripheral distribution [44]. Mitochondrial homeostasis is the basis of Rab7-mediated mitochondria–lysosome contacts, which contribute to the physiological division of mitochondria [45]. We uncover that Sod2, an indicator and regulator of mitochondrial homeostasis [46], can regulate lysosome positioning, which is consistent with promoting mitochondrial homeostasis induced by Sod2 and interaction with lysosomes.

We must highlight certain limitations in our study, although they do not detract our main conclusions. First, the specific advantages of GEV*^Sod2^* over other EV types and the interplay between Sod2 and lysosome positioning require further investigation. These areas will form the focus of our future studies, aiming to enhance the management of air pollution-related cardiopulmonary dysfunction. We performed DHE staining, 3-NT assay, and GSH/GSSG in vitro to evaluate the level of oxidative injury, which would be more appropriate to test additional indicators such as 8-oxoguanine and malondialdehyde as important complements [47]. In summary, our evaluation of the effectiveness of EVs extracted from human plasma, milk, and grapes in delivering a *Sod2*-overexpressing plasmid for treating PM2.5-induced cardiopulmonary injury indicates a distinct advantage of GEV*^Sod2^* in reducing cellular and tissue damage. Surprisingly, GEV*^Sod2^* influenced lysosome positioning in the PM2.5-exposed model, potentially affecting EV secretion. Our study offers a comparative analysis of the properties, biological performance, drug delivery potential, and storage forms of animal- versus plant-derived nanoparticles, enhancing the application of EVs in cardiopulmonary disease treatment.

## Materials and Methods

### Animals and PM2.5 exposure

Adult C57BL/6J male mice, 8 weeks old, were acquired from Charles River Laboratories (Beijing, China) and maintained under a 12-h light–dark cycle with access to a standard chow diet. Prior to PM2.5 exposure, mice were anesthetized with isoflurane for 3 min. Subsequently, the mice were administered 10 μl of PM2.5 solution via intratracheal instillation every other day for 1 week, as previously outlined [48]. Control mice received 10 μl of sterile PBS instead. For EV treatment, mice were anesthetized similarly and subsequently administered 10 μl of EV solution (the EV powder was first dissolved in PBS to a concentration of 40 μg/ml via intratracheal instillation of 24 h prior to PM2.5 exposure). After the completion of the model establishment, mice were allowed 24 h for recovery before undergoing bronchoalveolar lavage while being under anesthesia. The lung and heart tissues were then collected for subsequent analyses. All procedures involving animals and sample collection were performed in accordance with guidelines and were approved by the Ethics Committee of Shanghai University.

### Combination of bibliometric and bioinformatic analysis

To identify critical genes involved in PM2.5-induced cardiopulmonary injury, a “2-step” method incorporating bibliometric and bioinformatic analyses was employed. Bibliometric analysis was conducted to identify keywords with the most significant emergence and then to filter public sequencing data based on these results, followed by bioinformatic analysis to identify the most differentially expressed genes. We used the Web of Science Core collection to compile articles published from 2000 to 2022, using the following search terms: TS=“particulate matter” OR “air pollution” AND TS=“lung” OR “heart” OR “cardiovascular” OR “pulmonary” AND TS=“exosomes” OR “extracellular vesicles” OR “nanoparticle”. A total of 294 articles, including both research and review articles, were deemed relevant (Fig. S5). Records were exported in plain text format with full record and cited references, and bibliometrics analysis was conducted using an online platform (https://bibliometric.com/). Bioinformatics analysis compared the expression of classic anti-oxidative enzymes between air-filtered and PM2.5-exposed mice based on previously published data (PRJNA540011). Heat maps were generated.

### EV isolation and characterization

Following the guidelines of the Minimal Information for Studies of Extracellular Vesicles 2023 (MISEV2023) [49], we isolated EVs from healthy young human plasma, skimmed milk, and grapes (Vitis Vinifera “Summer Black”). EV extraction from bovine milk and plasma followed methodologies from previous studies [50] and our own work [51]. Grapes were processed as described in a prior study [40] by washing, peeling, removing seeds, and juicing. The juice was then centrifuged at increasing speeds (1,000*g* for 10 min, 2,000*g* for 20 min, 4,000*g* for 30 min, and 10,000*g* for 60 min) and filtered through a 0.22-μm membrane. The filtrate was subjected to ultracentrifugation at 100,000*g* for 90 min. The pellet was resuspended in PBS and further purified by sucrose density gradient centrifugation (8%, 30%, 45%, and 60%) at 100,000*g* for 120 min.

To characterize the isolated EVs, TEM, nanoparticle tracking analysis, and Western blotting were employed, as previously described [48]. For TEM, 10 μl of EVs was placed on a copper grid and left to settle for 2 min before air drying. EVs were then stained with 10 μl of uranyl acetate dihydrate for 2 min and air-dried again before TEM observation at 670,000× magnification. The relative number and size of EVs were determined by Zetaview ×30 (Particle Metrix, Germany), as previously described [51]. In addition, the presence of EV-specific protein markers, including CD9/CD63 and HSP70/TSPAN8, was assessed to confirm the isolation of EVs from plasma, milk, and grapes, respectively.

### Cell culture and treatment

To assess the effects of EVs on PM2.5-induced cell toxicity in vitro, human bronchial epithelioid cells, primary NRCMs, and human cardiomyocytes AC16 were cultured. Specifically, the protocol for NRCM isolation was adapted from a previous study [51,52]. For PM2.5 exposure and EV treatment, cells were pretreated with EVs at a concentration of 40 μg/ml for 24 h prior to PM2.5 exposure (50 μg/ml).

### FUGW-Sod2-overexpressing plasmid

The Sod2 gene from *Homo sapiens* (human) was subcloned into the FUGW vector (a gift from D. Baltimore, Addgene plasmid #14883[53]). The sequences of the primers used for plasmid construction are as follows: forward, 5′-CCGGAATTCATGTTGAGCCGGGCAGT-3′; reverse, 5′-CCGCTCGAGTTACTTTTTGCAAGCCATG-3′.

### DHE staining

Cells and tissue sections were subjected to DHE staining (#KGAFO19, Keygen Biotech Corp. Ltd., China) to assess levels of ROS. For cellular DHE staining, cells were washed with PBS and then incubated with DHE at a concentration of 1 μM for 30 min. After washing the stained cells, fluorescence imaging was performed using a Leica DMi8 microscope (Leica Microsystems, Germany). For tissue staining, heart or lung tissues were sectioned into 5-μm-thick frozen slices and allowed to equilibrate to room temperature for 20 min followed by 3 washes. The sections were subsequently incubated with DHE (30 μM) for 30 min. After 3 washes, the slices were mounted with antifade mounting medium and subjected to fluorescence imaging (Olympus, Japan).

### GSH/GSSG ratio

Cells and tissues were processed for GSH/GSSG detection using a commercial assay kit according to the manufacturer’s instructions (S0053, Beyotime Biotechnology, China). The total glutathione and GSSG levels were measured at 412 nm, and the GSH content, as well as its ratio to GSSG, was calculated according to the provided formula.

### 3-Nitrotyrosine

The 3-NT levels in tissues were determined using a 3-NT ELISA assay kit (E-EL-0040c, Elabscience, Wuhan, China). Briefly, 10 mg of lung or heart tissue was homogenized in 90 μl of precooled PBS at 0 to 4 °C and then centrifugated for 5 to 10 min at 5,000*g* at 4 °C to collect the supernatant. Samples were diluted to an appropriate concentration and assayed according to the manufacturer’s instructions. Data were normalized to the weight of the tissue used.

### H&E staining

Paraffin sections were subjected to H&E staining. Initially, sections were de-paraffinized using xylene and then rehydrated through a graded alcohol series before being thoroughly rinsed in water. The slides were stained with hematoxylin followed by a rinse in water and then counterstained with eosin. After staining, the slides were dehydrated using a graded series of alcohol and cleared with xylene before being mounted with glass coverslips for subsequent microscopy.

### Western blot and RNA quantification

Protein samples were prepared by lysing tissue or EVs in lysis buffer followed by centrifugation at 12,000*g* for 20 min. The supernatants were mixed with loading buffer and subjected to boiling lysis for 10 min. Protein samples (10 to 20 μg) were loaded for sodium dodecyl sulfate–polyacrylamide gel electrophoresis (SDS-PAGE) electrophoresis. The primary antibodies (1:1,000) used were against CD63 (catalog no. A5271, RRID:AB_2766092), HSP70 (no. A20819), TSPAN8 (catalog no. A13266, RRID:AB_2760119), Bax (catalog no. A12009, RRID:AB_2861644), Bcl2 (no. A20777), caspase-3 (no. A11040), Sod2 (catalog no. A19576, RRID:AB_2862677), and β-actin (catalog no. AC004, RRID:AB_2737399) from ABclonal Technology Co. Ltd. (China); calnexin (catalog no. 2679, RRID:AB_2228381) and CD9 (catalog no. 98327, RRID:AB_3351665) were from Cell Signaling Technology. Total RNA was isolated from tissues and cells using TRIzol Reagent. cDNA synthesis was performed using the RevertAid First Strand cDNA Synthesis Kit (Thermo Fisher Scientific, 01156807, USA), and mRNA levels were quantified by real-time PCR with SYBR Green Supermix (Bio-Rad, 172-5125, USA) on a Roche LightCycler480 PCR System as per our previous study [54]. Primers for real-time PCR were listed in Table S1, with 18*S* ribosomal RNA (rRNA) serving as the internal control for normalization.

### DiD staining and cellular uptake of EV assessment

EVs were labeled with DiD for fluorescence imaging. Initially, EVs were incubated with DiD dye for 30 min in the dark. Following incubation, the mixture was subjected to ultracentrifugation at 100,000*g* for 70 min to remove excess dye. The labeled EVs were then resuspended in cell culture medium and added to the cells for a 2-h treatment. Finally, the cells were fixed with 4% paraformaldehyde for 30 min, washed with PBS, and stained with Hoechst dye prior to fluorescence imaging.

### Sterility assays and mycoplasma detection

To assess the sterility of the EVs, solid Luria–Bertani (LB) medium plates (antibiotic-free) were inoculated with either EV powder or solution. The plates were then incubated at 37 °C for 14 d, after which colony formation was evaluated to determine sterility.

A one-step mycoplasma detection kit (40612ES25, Yeasen) was employed to detect mycoplasma, following the manufacturer’s instructions. The EV powder or solution, along with a positive control, was mixed with the reaction liquid and coloring solution and incubated at 30 °C for 5 min. The mixture was then transferred to a Bio-Rad C1000Touch thermocycler and incubated at 63 °C for 60 min. The color change of the solution was observed to determine the presence or absence of mycoplasma.

### High-sensitivity structured illumination microscopy

To analyze lysosome positioning, HIS-SIM (Guangzhou CSR Biotech Co. Ltd., China) was utilized for super-resolution imaging based on prior studies [55]. Beas-2B cells were seeded into μ-Slide 8 Well ibiTreat plates (#80826, ibidi GmbH, Germany) and cultured at 37 °C with 5% CO_2_ until reaching appropriate confluence. Cells were pretreated with DiD-labeling GEV^*Sod2*^ or control, followed by PM2.5 exposure for 24 h. Prior to HIS-SIM live imaging, cells were incubated with 50 nM LysoTracker Green (C1047S, Beyotime, China) for 1 h and then washed twice with PBS. Images were acquired using a 100×/1.5 NA (numerical aperture) oil immersion objective (Olympus), and sparse deconvolution was applied to enhance image quality.

### Quantification and statistical analysis

All data are presented as mean ± SD. After confirming normality assumptions (Shapiro–Wilk test) and homoscedasticity (*F* test), 2-tailed Student’s *t* test, 1-way analysis of variance (ANOVA), or 2-way ANOVA followed by Tukey’s multiple comparison test was used to analyze differences among multiple groups, depending on the specific experiment. All statistical graphs were generated using GraphPad Prism 8. A *P* value of less than 0.05 was considered statistically significant and *P < 0.05, **P < 0.01,***P < 0.001, ns *P* > 0.05.

## Data Availability

Data will be made available upon reasonable request.

## References

[1] Jin X, Yu H, Wang B, Sun Z, Zhang Z, Liu QS, Zheng Y, Zhou Q, Jiang G. Airborne particulate matters induce thrombopoiesis from megakaryocytes through regulating mitochondrial oxidative phosphorylation. Part Fibre Toxicol. 2021;18(1):19. 33985555 10.1186/s12989-021-00411-4 PMC8117637

[2] Shi L, Zanobetti A, Kloog I, Coull BA, Koutrakis P, Melly SJ, Schwartz JD. Low-concentration PM2.5 and mortality: Estimating acute and chronic effects in a population-based study. Environ Health Perspect. 2016;124(1):46–52. 26038801 10.1289/ehp.1409111 PMC4710600

[3] Yu W, Guo Y, Shi L, Li S. The association between long-term exposure to low-level PM2.5 and mortality in the state of Queensland, Australia: A modelling study with the difference-in-differences approach. PLOS Med. 2020;17(6): Article e1003141. 32555635 10.1371/journal.pmed.1003141 PMC7302440

[4] Christidis T, Erickson AC, Pappin AJ, Crouse DL, Pinault LL, Weichenthal SA, Brook JR, van Donkelaar A, Hystad P, Martin RV, et al. Low concentrations of fine particle air pollution and mortality in the Canadian community health survey cohort. Environ Health. 2019;18(1):84. 31601202 10.1186/s12940-019-0518-y PMC6785886

[5] An R, Kang H, Cao L, Xiang X. Engagement in outdoor physical activity under ambient fine particulate matter pollution: A risk-benefit analysis. J Sport Health Sci. 2022;11(4):537–544. 33035708 10.1016/j.jshs.2020.09.008 PMC7537654

[6] Wang H, Shen X, Liu J, Wu C, Gao J, Zhang Z, Zhang F, Ding W, Lu Z. The effect of exposure time and concentration of airborne PM_2.5_ on lung injury in mice: A transcriptome analysis. Redox Biol. 2019;26:101264. 31279222 10.1016/j.redox.2019.101264 PMC6612658

[7] Feng Y, Castro E, Wei Y, Jin T, Qiu X, Dominici F, Schwartz J. Long-term exposure to ambient PM2.5, particulate constituents and hospital admissions from non-respiratory infection. Nat Commun. 2024;15(1):1518. 38374182 10.1038/s41467-024-45776-0 PMC10876532

[8] Li Z, Hu S, Cheng K. Chemical engineering of cell therapy for heart diseases. Acc Chem Res. 2019;52(6):1687–1696. 31125198 10.1021/acs.accounts.9b00137 PMC7045701

[9] Lai Y, Cheng K, Kisaalita W. Three dimensional neuronal cell cultures more accurately model voltage gated calcium channel functionality in freshly dissected nerve tissue. PLOS ONE. 2012;7(9): Article e45074. 23049767 10.1371/journal.pone.0045074 PMC3458113

[10] Xie Y, Ibrahim A, Cheng K, Wu Z, Liang W, Malliaras K, Sun B, Liu W, Shen D, Cheol Cho H, et al. Importance of cell-cell contact in the therapeutic benefits of cardiosphere-derived cells. Stem Cells. 2014;32(9):2397–2406. 24802280 10.1002/stem.1736 PMC4138271

[11] Yates AG, Pink RC, Erdbrügger U, Siljander PR, Dellar ER, Pantazi P, Akbar N, Cooke WR, Vatish M, Dias-Neto E, et al. In sickness and in health: The functional role of extracellular vesicles in physiology and pathology in vivo: Part II: Pathology. J Extracell Vesicles. 2022;11(1): Article e12190. 35041301 10.1002/jev2.12190 PMC8765328

[12] Lou J, Wu J, Feng M, Dang X, Wu G, Yang H, Wang Y, Li J, Zhao Y, Shi C, et al. Exercise promotes angiogenesis by enhancing endothelial cell fatty acid utilization via liver-derived extracellular vesicle miR-122-5p. J Sport Health Sci. 2022;11(4):495–508. 34606978 10.1016/j.jshs.2021.09.009 PMC9338338

[13] Herrmann IK, Wood MJA, Fuhrmann G. Extracellular vesicles as a next-generation drug delivery platform. Nat Nanotechnol. 2021;16(7):748–759. 34211166 10.1038/s41565-021-00931-2

[14] Zhao B, Lin H, Jiang X, Li W, Gao Y, Li M, Yu Y, Chen N, Gao J. Exosome-like nanoparticles derived from fruits, vegetables, and herbs: Innovative strategies of therapeutic and drug delivery. Theranostics. 2024;14(12):4598–4621. 39239509 10.7150/thno.97096 PMC11373634

[15] Teng Y, He J, Zhong Q, Zhang Y, Lu Z, Guan T, Pan Y, Luo X, Feng W, Ou C. Grape exosome-like nanoparticles: A potential therapeutic strategy for vascular calcification. Front Pharmacol. 2022;13:1025768. 36339605 10.3389/fphar.2022.1025768 PMC9634175

[16] Hinderer C, Katz N, Buza EL, Dyer C, Goode T, Bell P, Richman LK, Wilson JM. Severe toxicity in nonhuman primates and piglets following high-dose intravenous administration of an adeno-associated virus vector expressing human SMN. Hum Gene Ther. 2018;29(3):285–298. 29378426 10.1089/hum.2018.015 PMC5865262

[17] Haapaniemi E, Botla S, Persson J, Schmierer B, Taipale J. CRISPR-Cas9 genome editing induces a p53-mediated DNA damage response. Nat Med. 2018;24(7):927–930. 29892067 10.1038/s41591-018-0049-z

[18] Ndeupen S, Qin Z, Jacobsen S, Estanbouli H, Bouteau A, Igyarto BZ. The mRNA-LNP platform’s lipid nanoparticle component used in preclinical vaccine studies is highly inflammatory. bioRxiv. 2021. 10.1101/2021.03.04.430128 PMC8604799 34841223

[19] Zhang A, Wang G, Jia L, Su T, Zhang L. Exosome-mediated microRNA-138 and vascular endothelial growth factor in endometriosis through inflammation and apoptosis via the nuclear factor-κB signaling pathway. Int J Mol Med. 2019;43(1):358–370. 30431056 10.3892/ijmm.2018.3980 PMC6257842

[20] Zhou QL, Bai YZ, Gao J, Duan Y, Lyu YC, Guan LF, Elkin K, Xie YL, Jiao Z, Wang HY. Human serum-derived extracellular vesicles protect A549 from PM 2.5-induced cell apoptosis. Biomed Environ Sci. 2021;34(1):40–49. 33531106 10.3967/bes2021.006

[21] Gorgens A, Corso G, Hagey DW, Jawad Wiklander R, Gustafsson MO, Felldin U, Lee Y, Bostancioglu RB, Sork H, Liang X, et al. Identification of storage conditions stabilizing extracellular vesicles preparations. J Extracell Vesicles. 2022;11(6): Article e12238. 35716060 10.1002/jev2.12238 PMC9206228

[22] Wei M, Bao G, Li S, Yang Z, Cheng C, Le W. PM2.5 exposure triggers cell death through lysosomal membrane permeabilization and leads to ferroptosis insensitivity via the autophagy dysfunction/p62-KEAP1-NRF2 activation in neuronal cells. Ecotoxicol Environ Saf. 2022;248:114333. 36446170 10.1016/j.ecoenv.2022.114333

[23] Li P, Wang X, Zhao M, Song R, Zhao KS. Polydatin protects hepatocytes against mitochondrial injury in acute severe hemorrhagic shock via SIRT1-SOD2 pathway. Expert Opin Ther Targets. 2015;19(7):997–1010. 26073907 10.1517/14728222.2015.1054806

[24] Jongsma ML, Bakker J, Cabukusta B, Liv N, van Elsland D, Fermie J, Akkermans JL, Kuijl C, van der Zanden SY, Janssen L, et al. SKIP-HOPS recruits TBC1D15 for a Rab7-to-Arl8b identity switch to control late endosome transport. EMBO J. 2020;39(6): Article e102301. 32080880 10.15252/embj.2019102301 PMC7073467

[25] Ding Y, Zhang R, Li B, Du Y, Li J, Tong X, Wu Y, Ji X, Zhang Y. Tissue distribution of polystyrene nanoplastics in mice and their entry, transport, and cytotoxicity to GES-1 cells. Environ Pollut. 2021;280:116974. 33784569 10.1016/j.envpol.2021.116974

[26] Abramson J, Adler J, Dunger J, Evans R, Green T, Pritzel A, Ronneberger O, Willmore L, Ballard AJ, Bambrick J, et al. Accurate structure prediction of biomolecular interactions with AlphaFold 3. Nature. 2024;630(8016):493–500. 38718835 10.1038/s41586-024-07487-w PMC11168924

[27] van de Wakker SI, van Oudheusden J, Mol EA, Roefs MT, Zheng W, Gorgens A, El Andaloussi S, Sluijter JPG, Vader P. Influence of short term storage conditions, concentration methods and excipients on extracellular vesicle recovery and function. Eur J Pharm Biopharm. 2022;170:59–69. 34864197 10.1016/j.ejpb.2021.11.012

[28] El Baradie KBY, Nouh M, O’Brien Iii F, Liu Y, Fulzele S, Eroglu A, Hamrick MW. Freeze-dried extracellular vesicles from adipose-derived stem cells prevent hypoxia-induced muscle cell injury. Front Cell Dev Biol. 2020;8:181. 32266262 10.3389/fcell.2020.00181 PMC7099601

[29] Collins J, Robinson C, Danhof H, Knetsch CW, van Leeuwen HC, Lawley TD, Auchtung JM, Britton RA. Dietary trehalose enhances virulence of epidemic Clostridium difficile. Nature. 2018;553(7688):291–294. 29310122 10.1038/nature25178 PMC5984069

[30] Sethi S. Is it the heart or the lung? Sometimes it is both. J Am Heart Assoc. 2022;11(18): Article e027112. 10.1161/JAHA.122.027112 PMC9683680 36102223

[31] Katira BH, Giesinger RE, Engelberts D, Zabini D, Kornecki A, Otulakowski G, Yoshida T, Kuebler WM, McNamara PJ, Connelly KA, et al. Adverse heart-lung interactions in ventilator-induced lung injury. Am J Respir Crit Care Med. 2017;196(11):1411–1421. 28795839 10.1164/rccm.201611-2268OC

[32] Dransfield MT, Criner GJ, Halpin DMG, Han MK, Hartley B, Kalhan R, Lange P, Lipson DA, Martinez FJ, Midwinter D, et al. Time-dependent risk of cardiovascular events following an exacerbation in patients with chronic obstructive pulmonary disease: Post hoc analysis from the IMPACT trial. J Am Heart Assoc. 2022;11(18): Article e024350. 36102236 10.1161/JAHA.121.024350 PMC9683674

[33] Li J, Sun S, Zhu D, Mei X, Lyu Y, Huang K, Li Y, Liu S, Wang Z, Hu S, et al. Inhalable stem cell exosomes promote heart repair after myocardial infarction. Circulation. 2024;150(9):710–723. 39186525 10.1161/CIRCULATIONAHA.123.065005 PMC11349039

[34] Popowski KD, López de Juan Abad B, George A, Silkstone D, Belcher E, Chung J, Ghodsi A, Lutz H, Davenport J, Flanagan M, et al. Inhalable exosomes outperform liposomes as mRNA and protein drug carriers to the lung. Extracell Vesicle. 2022;1:100002. 36523538 10.1016/j.vesic.2022.100002 PMC9213043

[35] van der Pol A, van Gilst WH, Voors AA, van der Meer P. Treating oxidative stress in heart failure: Past, present and future. Eur J Heart Fail. 2019;21(4):425–435. 30338885 10.1002/ejhf.1320 PMC6607515

[36] Scoditti E, Massaro M, Garbarino S, Toraldo DM. Role of diet in chronic obstructive pulmonary disease prevention and treatment. Nutrients. 2019;11(6):1357. 31208151 10.3390/nu11061357 PMC6627281

[37] Wang H, Shen X, Tian G, Shi X, Huang W, Wu Y, Sun L, Peng C, Liu S, Huang Y, et al. AMPKα2 deficiency exacerbates long-term PM_2.5_ exposure-induced lung injury and cardiac dysfunction. Free Radic Biol Med. 2018;121:202–214. 29753072 10.1016/j.freeradbiomed.2018.05.008

[38] Dolgin E. mRNA drug offers hope for treating a devastating childhood disease. Nature. 2024;628(8007):248. 38570656 10.1038/d41586-024-00954-4

[39] Kim J, Li S, Zhang S, Wang J. Plant-derived exosome-like nanoparticles and their therapeutic activities. Asian J Pharm Sci. 2022;17(1):53–69. 35261644 10.1016/j.ajps.2021.05.006 PMC8888139

[40] Ju S, Mu J, Dokland T, Zhuang X, Wang Q, Jiang H, Xiang X, Deng ZB, Wang B, Zhang L, et al. Grape exosome-like nanoparticles induce intestinal stem cells and protect mice from DSS-induced colitis. Mol Ther. 2013;21(7):1345–1357. 23752315 10.1038/mt.2013.64 PMC3702113

[41] Ivanova NN, Khomich LM, Perova IB, Eller KI. Grape juice nutritional profile. Vopr Pitan. 2018;87(6):95–105. 10.24411/0042-8833-2018-10046 30763495

[42] Pu J, Guardia CM, Keren-Kaplan T, Bonifacino JS. Mechanisms and functions of lysosome positioning. J Cell Sci. 2016;129(23):4329–4339. 27799357 10.1242/jcs.196287 PMC5201012

[43] Dodson MW, Zhang T, Jiang C, Chen S, Guo M. Roles of the drosophila LRRK2 homolog in Rab7-dependent lysosomal positioning. Hum Mol Genet. 2012;21(6):1350–1363. 22171073 10.1093/hmg/ddr573 PMC3284123

[44] Jia R, Bonifacino JS. Lysosome positioning influences mTORC2 and AKT signaling. Mol Cell. 2019;75(1):26–38.e3. 31130364 10.1016/j.molcel.2019.05.009 PMC7446307

[45] Wong YC, Ysselstein D, Krainc D. Mitochondria-lysosome contacts regulate mitochondrial fission via RAB7 GTP hydrolysis. Nature. 2018;554(7692):382–386. 29364868 10.1038/nature25486 PMC6209448

[46] Bolduc JA, Collins JA, Loeser RF. Reactive oxygen species, aging and articular cartilage homeostasis. Free Radic Biol Med. 2019;132:73–82. 30176344 10.1016/j.freeradbiomed.2018.08.038 PMC6342625

[47] Sies H, Berndt C, Jones DP. Oxidative stress. Annu Rev Biochem. 2017;86:86715–86748. 10.1146/annurev-biochem-061516-045037 28441057

[48] Wang H, Wang T, Rui W, Xie J, Xie Y, Zhang X, Guan L, Li G, Lei Z, Schiffelers RM, et al. Extracellular vesicles enclosed-miR-421 suppresses air pollution (PM(2.5) )-induced cardiac dysfunction via ACE2 signalling. J Extracell Vesicles. 2022;11(5): Article e12222. 35536587 10.1002/jev2.12222 PMC9089227

[49] Welsh JA, Goberdhan DCI, O’Driscoll L, Buzas EI, Blenkiron C, Bussolati B, Cai H, Di Vizio D, Driedonks TAP, Erdbrugger U, et al. Minimal information for studies of extracellular vesicles (MISEV2023): From basic to advanced approaches. J Extracell Vesicles. 2024;13(2): Article e12404. 38326288 10.1002/jev2.12404 PMC10850029

[50] Munagala R, Aqil F, Jeyabalan J, Gupta RC. Bovine milk-derived exosomes for drug delivery. Cancer Lett. 2016;371(1):48–61. 26604130 10.1016/j.canlet.2015.10.020 PMC4706492

[51] Wang H, Maimaitiaili R, Yao J, Xie Y, Qiang S, Hu F, Li X, Shi C, Jia P, Yang H, et al. Percutaneous intracoronary delivery of plasma extracellular vesicles protects the myocardium against ischemia-reperfusion injury in Canis. Hypertension. 2021;78(5):1541–1554. 34488435 10.1161/HYPERTENSIONAHA.121.17574

[52] Vandergriff AC, Hensley MT, Cheng K. Isolation and cryopreservation of neonatal rat cardiomyocytes. J Vis Exp. 2015;(98):52726. 25938862 10.3791/52726 PMC4541493

[53] Lois C, Hong EJ, Pease S, Brown EJ, Baltimore D. Germline transmission and tissue-specific expression of transgenes delivered by lentiviral vectors. Science. 2002;295(5556):868–872. 11786607 10.1126/science.1067081

[54] Gao J, Lei T, Wang H, Luo K, Wang Y, Cui B, Yu Z, Hu X, Zhang F, Chen Y, et al. Dimethylarginine dimethylaminohydrolase 1 protects PM2.5 exposure-induced lung injury in mice by repressing inflammation and oxidative stress. Part Fibre Toxicol. 2022;19(1):64. 36242005 10.1186/s12989-022-00505-7 PMC9569114

[55] Huang X, Fan J, Li L, Liu H, Wu R, Wu Y, Wei L, Mao H, Lal A, Xi P, et al. Fast, long-term, super-resolution imaging with hessian structured illumination microscopy. Nat Biotechnol. 2018;36(5):451–459. 29644998 10.1038/nbt.4115

