# Supplementary material for: GEVSod2 Powder: A Modified Product Based on Biovesicles Functioned in Air Pollution PM2.5-Induced Cardiopulmonary Injury
Source: Research (Wash D C). 2025 Feb 13;8:0609. doi: 10.34133/research.0609 (PMC11822167; doi:10.34133/research.0609)
Supplement: Supplementary 1 — Figs. S1 to S5 Table S1 [file research.0609.f1.docx]

**Supplementary data Fig. S1 to S5**

**
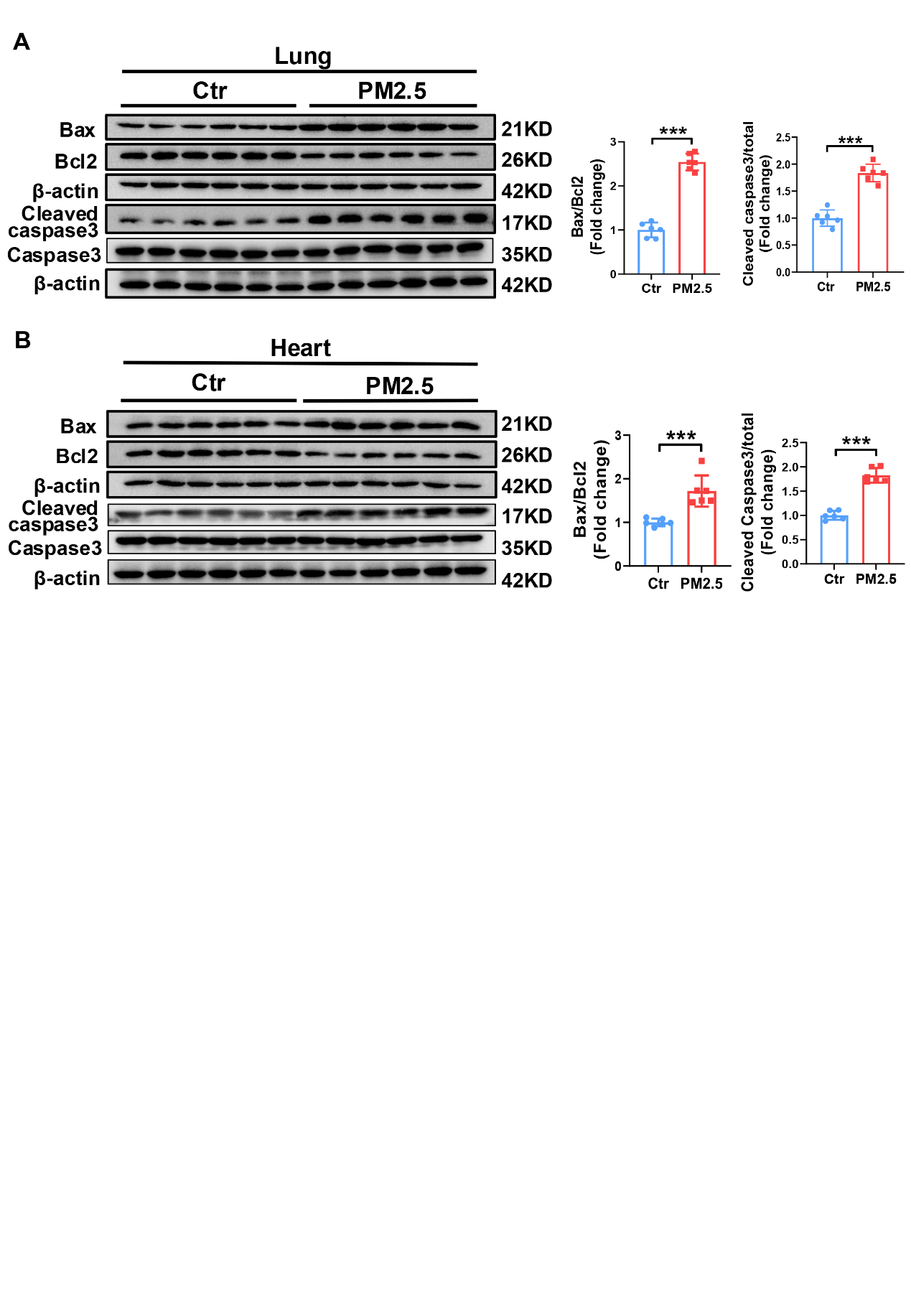
**

**Fig S1 Acute PM2.5 exposure causes cardiopulmonary cell apoptosis.** Adult male C57BL/6J mice were administered 10µL of PM2.5 by intratracheal instillation every other day for one week. Lung and heart tissues were harvested after the following examination. Expression of Bax, Bcl-2, cleaved caspase-3 and total caspase-3 protein were detected by western blotting and quantified in (A) lungs and (B) hearts (Ctr, n=6 versus PM2.5, n=6). Data were shown as means ± SD. P values were calculated with the unpaired, two-tailed Student's t test after passing normality test (A, B).


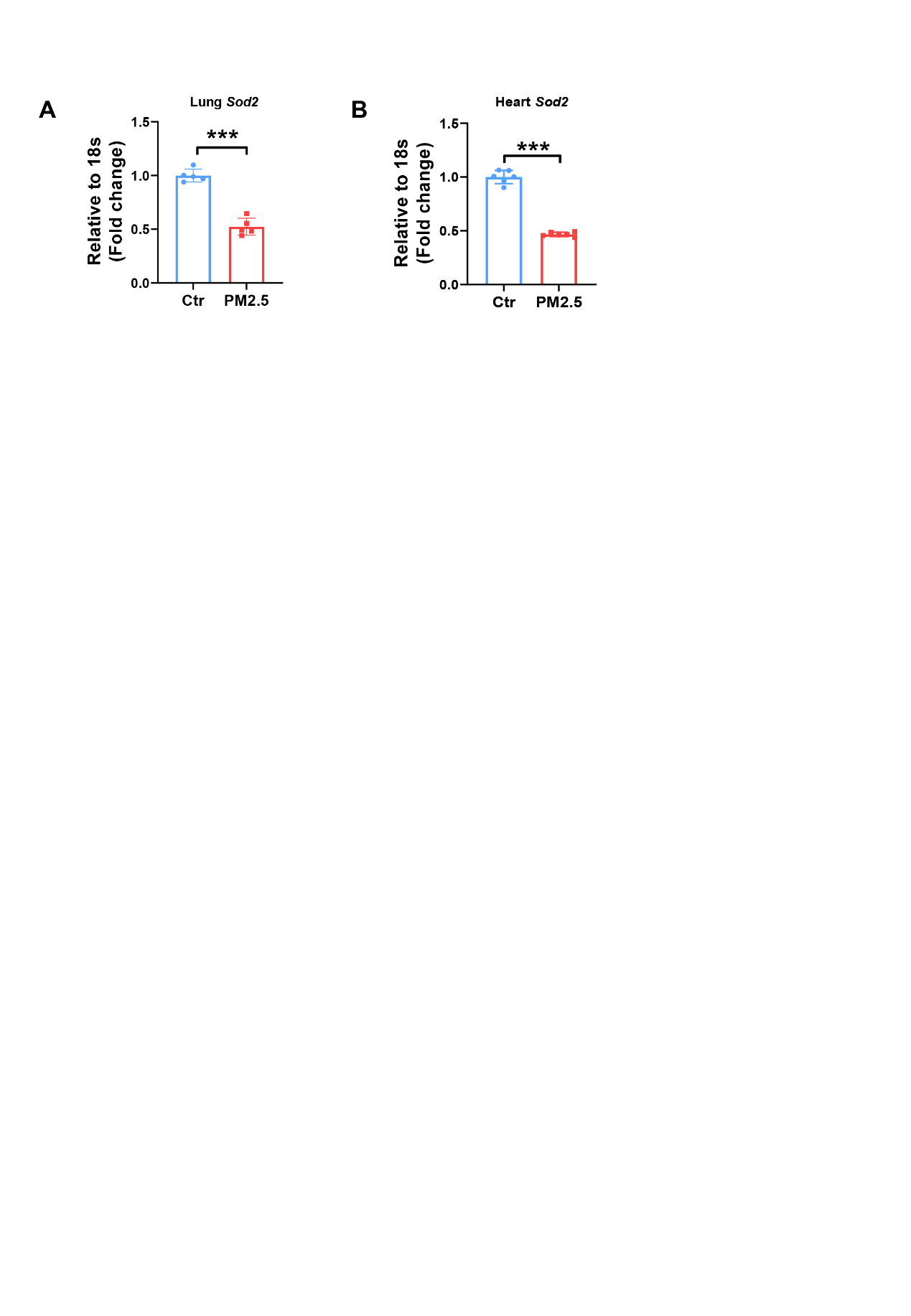


**Fig S2 PM2.5 exposure caused a reduction of Sod2 mRNA.** The mRNA level of Sod2 was tested in the Ctr- or PM2.5-exposed (A) lung tissues and (B) heart tissues. (Ctr, n=6 versus PM2.5, n=6). Data were shown as means ± SD. P values were calculated with the unpaired, two-tailed Student's t test after passing normality test (A, B).

**
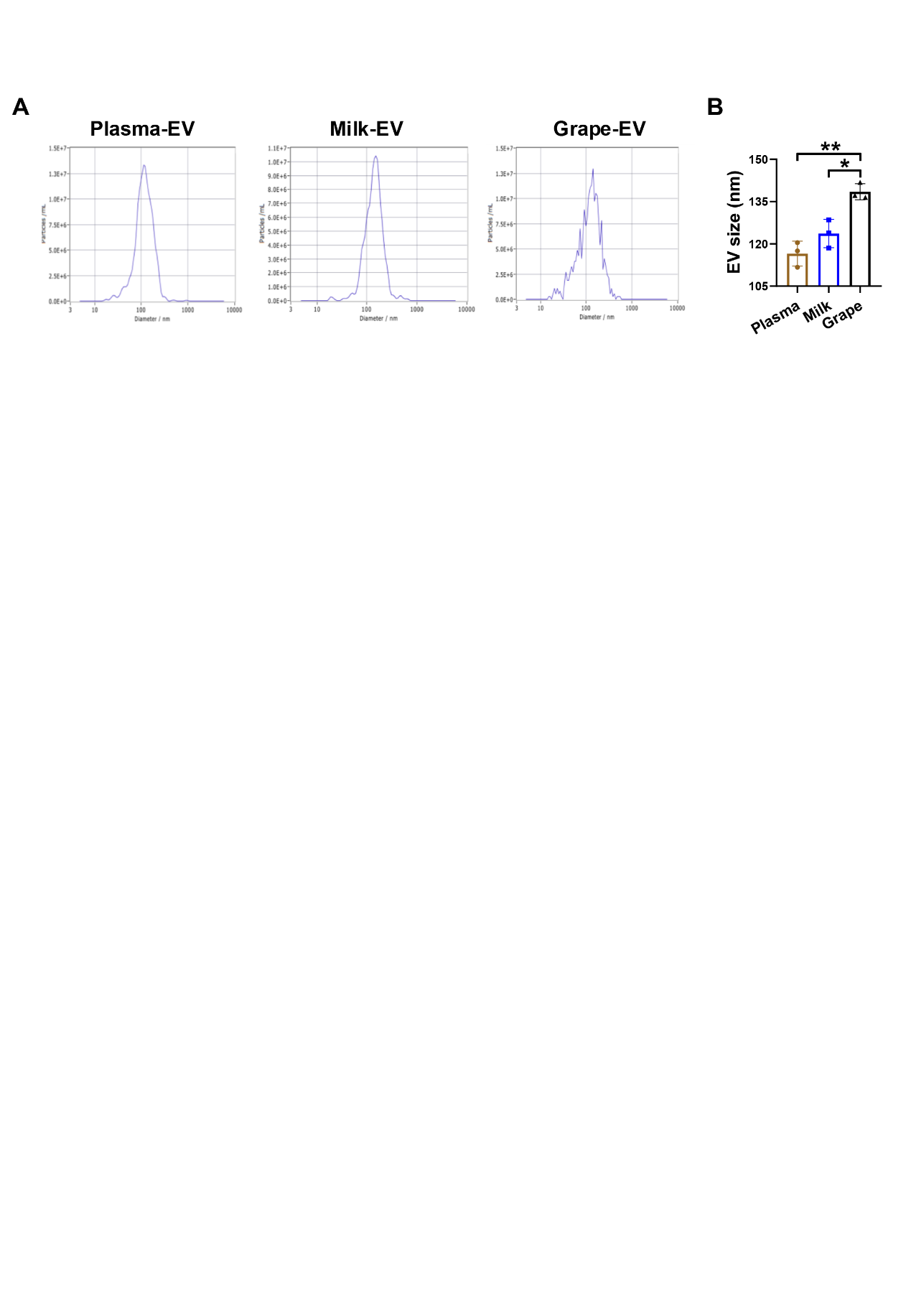
**

**Fig S3 The size of nanoparticles.** (A) Nanoparticle tracking analysis (NTA) of human plasma-, milk- and fresh grape juice-derived EVs were shown. (B) The size of EVs were collected based on NTA test (n=3).

**
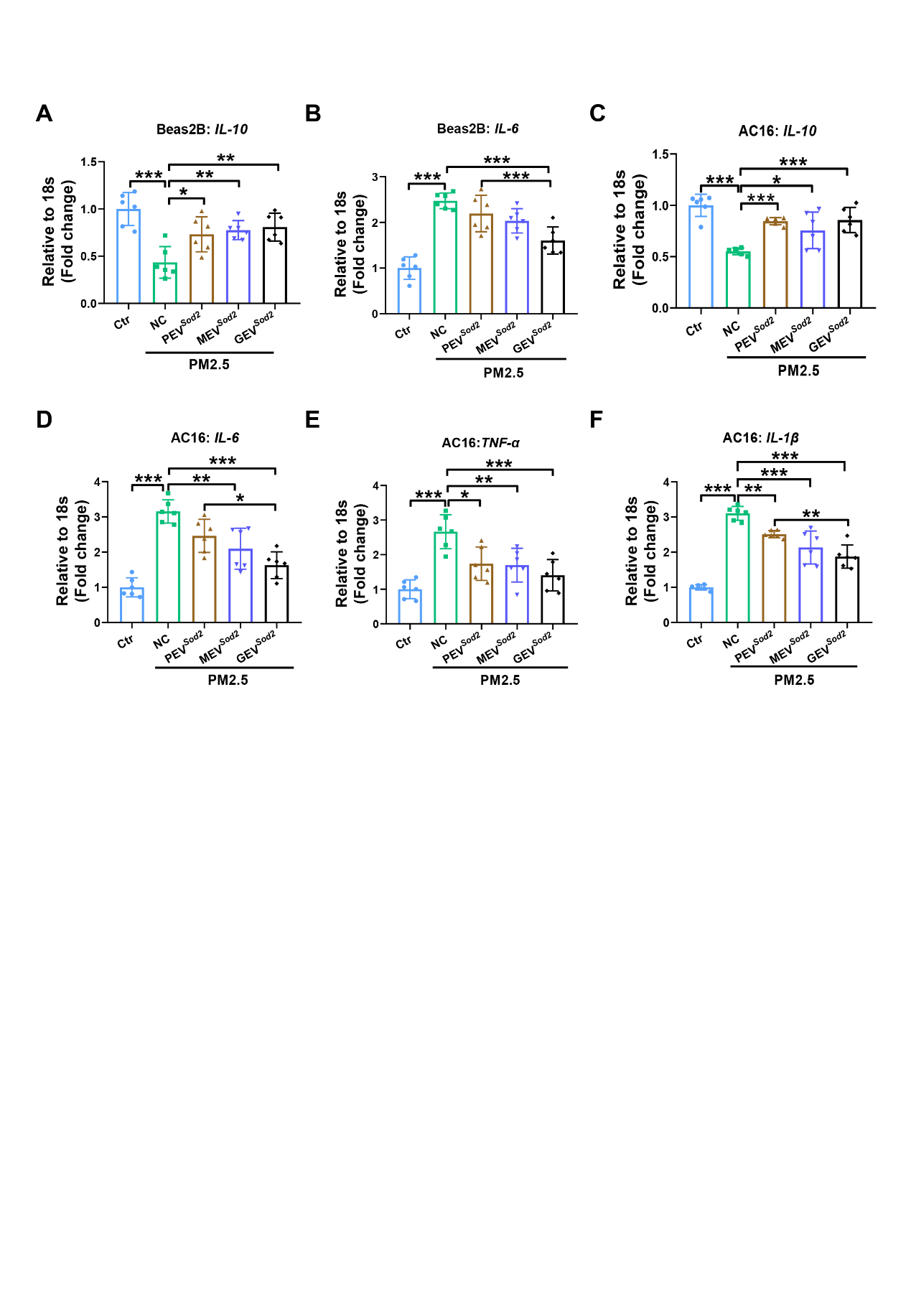
**

**Fig S4 The effect of PEV^Sod2^, MEV^Sod2^ and GEV^Sod2^ on the secretion of inflammatory factors in PM2.5-exposed model.** To assess whether the modified EV’s immunogenicity, the mRNA level of inflammatory factors, including IL-10, TNFα, IL-1β and IL-6 in PM2.5-challenged cell model were tested. (A-B) The IL-10 and IL-6 level of PM2.5-exposed model in Beas-2B cells (Ctr, n=6 versus n=6, n=6, n=6, n=6). (C-F) IL-10, IL-6, TNFα and IL-1β level of PM2.5-exposed model in human AC16 cells (Ctr, n=6 versus n=6, n=6, n=6, n=6). Data are shown as mean s± SD. To compare multiple groups, one-way ANOVA test with Tukey’s post hoc test was used.


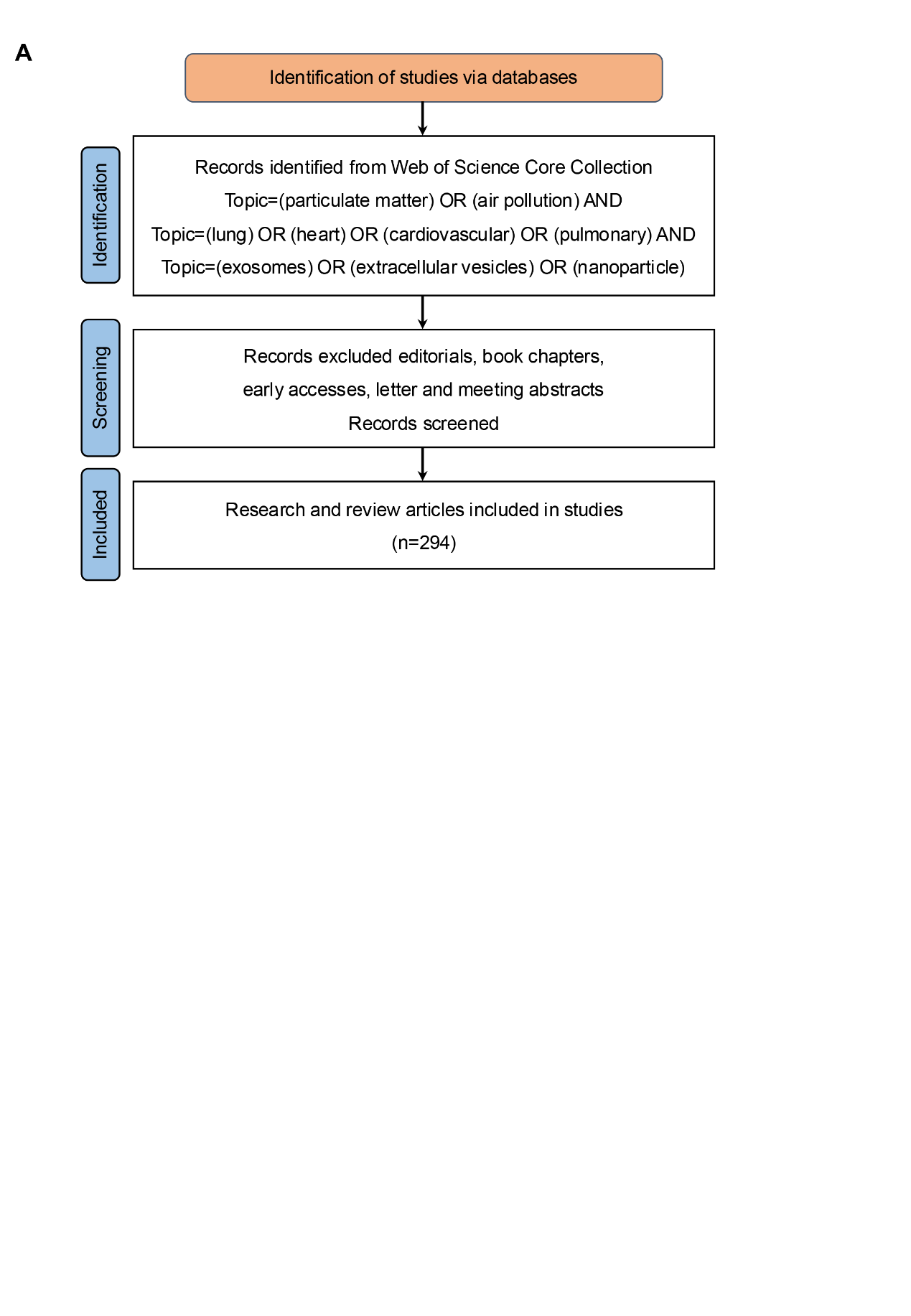


**Fig S5 The bibliometrics flow chart.** The flow chart showed details of bibliometrics analysis in this study.

**Supplementary data Tables S1**

**The primers for Real-time PCR**

| Species | Gene Name | Primer Sequence |
| --- | --- | --- |
| Mouse | *18s* | F: TCAAGAACGAAAGTCGGAGG |
|  |  | R: GGACATCTAAGGGCATCAC |
|  | *TNFα* | F: CCCTCACACTCAGATCATCTTCT |
|  |  | R: GCTACGACGTGGGCTACAG |
|  | *IL-6* | F: CCAAGAGGTGAGTGCTTCCC |
|  |  | R: CTGTTGTTCAGACTCTCTCCCT |
|  | *IL-1β* | F: GACGGACCCCAAAAGATGAAG |
|  |  | R: CTCTTCGTTGATGTGCTGCTGTG |
|  | *Sod2* | F: ATTGACGTGTGGGAGCA |
|  |  | R: AATGTGGCCGTGAGTGA |
| Human | *TNFα* | F: CTCGAACCCCGAGTGACAAG |
|  |  | R: TATCTCTCAGCTCCACGCCA |
|  | *IL-6* | F: AGTGAGGAACAAGCCAGAGC |
|  |  | R: AGCTGCGCAGAATGAGATGA |
|  | *IL-1β* | F: TGAGCTCGCCAGTGAAATGA |
|  |  | R: AGATTCGTAGCTGGATGCCG |
|  | *IL-10* | F: TGCCTGGTCCTCCTGACTG |
|  |  | R: CGAAGCATGTTAGGCAGGTTG |
|  | *Sod2* | F: CGACCTGCCCTACGACT |
|  |  | R: ACGCCTCCTGGTACTTCTC |
